# Revolutionizing root canal treatment: A review of minimally invasive endodontics

**DOI:** 10.6026/973206300212504

**Published:** 2025-08-31

**Authors:** Puneet Prasher, Simran Jyot Kaur, Mehar Kaur, Barbara Vigas, Tamana Khatri, Sairam Pallerla

**Affiliations:** 1 Department of Dental Surgery in Prosthodontics and Crown & Bridge, Sri Guru Ram Das Institute of Dental Sciences and Research, Punjab, India; 2 Department of Dental Surgery, Sibar Institute of Dental Sciences, Guntur, India; 3 Department of Dental Surgery, Sudha Rustagi College of Dental Sciences & Research, Faridabad, India; 4 Department of Dental Prosthetics, Faculdade do mazonas (IAES), Manaus, AM, Brazil; 5 Department of Dental Surgery, Dow University of Health Sciences, Karachi, Pakistan; 6 Department of Dental Surgery, Sri Sai College of Dental Surgery & Hospital, Vikarabad, Hyderabad, India

**Keywords:** Conservative access cavity, cone-beam computed tomography, endocrowns, micro-invasive irrigation protocols, bioceramic sealers

## Abstract

Minimally invasive endodontics (MIE) is an emerging approach in root canal therapy that emphasizes preserving as much tooth structure as possible while maintaining effective disinfection, which is essential for achieving long-term clinical success. Therefore, it is of interest to review the biomechanical and clinical outcomes of minimally invasive endodontics (MIE) in comparison to conventional endodontic techniques, while also addressing the efficacy of instrumentation, canal debridement and fracture resistance within these approaches. Additionally, emerging treatment modalities such as three-dimensional guided endodontics and enhanced irrigation protocols have been explored. Data shows that MIE is a promising strategy for preserving both the form and function of the tooth. However, further clinical studies are needed to standardize protocols and confirm long-term success rates.

## Background:

Minimally Invasive Endodontics (MIE) marks a significant transformation in how root canal treatments are approached. Its core objective is to preserve as much natural tooth structure as possible while effectively eliminating infection, disinfecting the root canal system and preventing reinfection. In contrast to older practices that relied heavily on wide access cavities and extensive canal shaping, MIE emphasizes more targeted approaches that safeguard the tooth's integrity [1]. This evolution mirrors the broader shift in dentistry toward preservation-focused care. Techniques in MIE incorporate reduced access preparation, minimal instrumentation and modern irrigation protocols to conserve critical structures such as pericervical dentin, which are essential for maintaining tooth strength and function. The approach aims to balance adequate debridement and the conservation of sound dentin, particularly in the cervical region [2]. Recent open-access studies confirm that excessive dentin removal correlates with a heightened risk of fractures and long-term restorative failure. Conversely, minimally invasive approaches have been shown to enhance fracture resistance, improve marginal integrity and support durable adhesive restorations. In the past, conventional access designs were dictated by limitations in visualization and instrumentation, often requiring substantial removal of coronal tooth structure. However, modern endodontic tools such as rotary NiTi files, cone-beam computed tomography (CBCT) and high-powered dental microscopes enable clinicians to navigate canals with significantly less structural loss [1, 3]. Current research supports using contracted or conservative access cavities, which allow for sufficient cleaning and shaping while maintaining more of the tooth's original strength and structure [4, 5].

## Technological and clinical advances enabling MIE:

## Clinically predictable MIE:

[1] CBCT Imaging and 3D Navigation: Enable accurate visualization and guided conservative access, minimizing unnecessary dentin removal.

[2] Dental operating microscopes: Enhance canal detection under magnification and illumination while preserving tooth structure.

[3] Heat-treated NiTi rotary instruments: Increase flexibility and durability, allowing safe shaping through restricted access cavities.

[4] Ultrasonic irrigation systems (*e.g.*, Gentle Wave^TM^): Promote efficient canal disinfection without aggressive canal enlargement [3].

[5] Bioceramic sealers: Support obturation of minimally shaped canals by offering superior sealing properties [5].

These advances have shifted MIE from a theoretical ideal into a viable, everyday clinical strategy.

## Access cavity preparation in minimally invasive endodontics:

Minimally invasive endodontics (MIE) has revolutionized the approach to root canal treatments, prioritizing the preservation of healthy tooth structure to enhance long-term tooth survival. Access cavity preparation, a critical first step in endodontic therapy, has evolved from traditional, extensive designs to conservative techniques supported by cutting-edge technology.

## Traditional vs. Minimally invasive access cavities:

Trad AC has prioritized straight-line access to the root canal system and demanded significant coronal tooth structure loss, including the pulp chamber roof. This approach sought to promote instrumentation and irrigation but heavily depended on the tooth's structural integrity and was prone to promoting post-treatment fractures [6]. The shift toward MIAC focuses on preserving paracervical dentin and enamel, which are critical for stress distribution and fracture resistance.

## MIAC designs include:

[1] Conservative access cavity (ConsAC): Partial deroofing of the pulp chamber while retaining pulp horns and convergent walls [7].

[2] Ultraconservative access cavity (UltraAC): A "ninja" or point-access design that minimizes occlusal extension, preserving maximal tooth structure [8].

[3] Truss access cavity (TrussAC): These are separate cavities for canal orifices, maintaining a dentinal bridge between them [9].

## Technological advancements:

[1] Magnification and illumination: Using the dental operating microscope and loupes allows better visibility in the restricted access cavities, leading to better canal detection and fewer procedural errors.

[2] 3D Imaging and guided endodontics: CBCT-guided static templates and dynamic navigation systems (*e.g.*, the X-NAV System) present 3D-printed stents or real-time tracking, enabling precision-directed burs through complex canals. CBCT combined with dynamic navigation systems allows accurate access cavity planning and performance, especially in difficult anatomical conditions [10].

[3] Introduction of Nickel-Titanium (NiTi) Instruments: The heat-treated NiTi files showed superior cyclic fatigue resistance in curved canals, enabling smaller access preparations while providing safer shaping.

[4] Ultrasonic Tips: Ultrasonic tips, like diamond-coated ET18D, allow controlled dentin cutting and pulp stone removal, thus reducing damage to the pulp chamber floor. These advances improve outcomes but necessitate skilled staff and expensive equipment and their access is somewhat limited in certain practices [11].

## Clinical implications and evidence:

[1] Fracture resistance: Studies comparing TradAC and MIAC report mixed findings. While some suggest that ConsAC and TrussAC improve fracture resistance in teeth with intact marginal ridges, others found no significant difference when marginal ridges were lost. UltraAC showed no mechanical benefits added to ConsAC.

[2] Canal detection and cleaning efficacy.

[3] Pros: ConsAC provides other debridement equivalents to TradAC in straight canals. Cons: UltaAC and TrussAC: Missed canals (*e.g.*, MB2 in molars) and poor irrigation effectiveness because of restricted access.

[4] Procedural challenges: Cyclic fatigue: Reduced straight-line access angles can increase rotary instruments' torsional stress, decreasing longevity.

[5] Sealing issues: Voids in root canal obturations and remaining pulp chamber debris are more frequent with UltraAC.

## Minimally invasive shaping techniques in endodontics:

The principle of minimally invasive shaping aims to clean the root canal system while efficiently protecting dentin. It relies on respecting the original anatomy of the canals using conservative access cavities, low-taper instruments and less invasive approaches [12]. Systems like Trunatomy, XP Endo-Shaper HyFlex EDM and SAF align with this philosophy and represent innovations that seek to improve treatment outcomes while reducing structural tooth damage.

[1] TruNatomy system: Developed by Dentsply Sirona in 2019, truNatomy is a rotary system specifically designed to maintain the natural anatomy of the root canal while minimizing unnecessary dentin removal [13]. Its main features are its reverse taper and reduced maximum diameter (0.08mm), which helps protect peri cervical dentin, essential to the tooth's long-term survival and structural integrity [14]. The files are manufactured using a heat-treated nickel-titanium alloy, providing increased flexibility and cyclic fatigue resistance. TruNatomy has been shown to produce less canal transportation when compared to other file systems, such as Protaper Gold [15].

[2] XP endo-shaper: The XP-Endo shaper is a single-file system made of MaxWire alloy, a nickel-titanium material that changes shape with temperature. When exposed to body warmth, the file expands and adapts to the canal morphology, reaching areas typically untouched by conventional instruments. This adaptive behavior allows improved contact with canal walls, enhancing debridement while retaining the canal's original anatomy.

[3] Hyflex EDM: The HyFlex EDM system introduced by Coltène incorporates electrical discharge machining (EDM) technology, a manufacturing process that uses controlled electrical discharges instead of direct contact. This results in a surface with fewer microcracks and significantly higher resistance to cyclic fatigue, up to 700% greater than traditional NiTi files. The files also feature a variable cross-sectional design from CM-Wire, which provides controlled memory. In some cases, this memory allows the files to recover their original shape after sterilization, making them suitable for reuse [16].

[4] SAF: The SAF system by ReDent Nova uses a hollow, compressible NiTi mesh that adapts to the canal shape during movement. Its structure shrinks or expands according to the canal anatomy, promoting gentle, uniform cleaning while preserving dentin. Its built-in irrigation system delivers a continuous flow of irrigants, improving disinfection without pressure accidents [17].

[5] Non-instrumentation endodontics (NIE): NIE proposes root canal cleaning with minimal mechanical shaping, relying on advanced irrigation. The GentleWave system exemplifies this approach by using multisonic acoustic energy and controlled fluid flow to clean areas untouched by files. It improves disinfection while preserving tooth structure, representing a promising step in minimally invasive endodontics [18].

## Advancements in irrigation and disinfection:

Minimally invasive endodontics (MIE) prioritizes preserving tooth structure while ensuring effective root canal disinfection. New findings on irrigation systems, irrigants, disinfecting agents and new technologies help achieve cleaning effectiveness even in challenging canal architectures and are consistent with MIE's conservation philosophy.

## Enhanced irrigation techniques:

Apical negative pressure irrigation, exemplified by the EndoVac system (Kerr Dental), delivers irrigants to the apex via a microcannula (0.55 mm) and a microcannula (0.32 mm), minimizing extrusion risks and removing debris effectively in minimally shaped canals (apical size ≥35). Studies show EndoVac achieves cleaner apical thirds than syringe irrigation [19]. The GentleWave system (Sonendo) employs ultrasonic ultra-cleaning, generating acoustic cavitation and vortical flow to clean canals with minimal instrumentation (apical size 15-25). It dissolves tissue eight times faster than ultrasonic systems, enhancing biofilm removal [20].

## Laser-Activated irrigation:

Photon-induced photoacoustic streaming (PIPS) and Shock Wave Enhanced Emission Photoacoustic Streaming (SWEEPS), using Erbium: YAG lasers (*e.g.*, LightWalker®), agitate irrigants in the pulp chamber, producing turbulent streaming without requiring deep canal access. A PIP achieves 100% Enterococcus faecalis inhibition in 20 seconds with 6% sodium hypochlorite, ideal for conservative preparations [21]. SWEEPS enhances cleaning by emitting dual laser pulses, accelerating bubble collapse and shock wave emission and improving smear layer removal in narrow canals [22].

## Passive ultrasonic irrigation:

Passive ultrasonic irrigation (PUI) employs ultrasonic files (25-30 kHz) to generate acoustic streaming and cavitation, enhancing irrigant penetration into uninstrumented areas. PUI removes pulpal remnants and smear layers more effectively than syringe irrigation, requiring minimal canal enlargement (apical size 30-35), aligning with MIE principles [23].

## Advanced irrigation solutions:

Enhanced sodium hypochlorite (NaOCl, 0.5-6%) formulations remain the gold standard for tissue dissolution and antimicrobial action, though limited by smear layer removal. Nanoparticle-based irrigants, such as silver, zinc oxide and chitosan, disrupt biofilms with high antimicrobial efficacy but may leave precipitates [24]. Chelators like EDTA and surfactants (*e.g.*, MTAD, QMix) effectively remove smear layers and enhance NaOCl penetration, supporting cleaner canals in MIE [25].

## Advanced disinfection agents:

Photodynamic therapy (PDT) uses photosensitizers (*e.g.*, methylene blue) and light to target microbial cells, reducing postoperative pain. Ozone therapy, delivered as gas or ozonated water, provides broad-spectrum antimicrobial effects, though its long-term efficacy requires further validation. Cold atmospheric plasma (CAP) is an emerging non-thermal disinfection method that disrupts biofilms through reactive species while being biocompatible. These adjunctive therapies enhance disinfection in minimally invasive cases with restricted mechanical access [23].

## Emerging technologies:

Customized irrigation devices printed in 3D, specifically for the patient's anatomy of canals, are being experimented with. Still, clinical data is lacking to support their use and research is based on prototype efficiency [24].

## Clinical relevance and practical applications:

These developments allow complete disinfection without removing much dentin, maintaining tooth structure and reducing fracture incidence in MIE. However, high costs, steep learning curves and extended armamentarium requirements hinder routine adoption. Techniques like GentleWave and PIPS show promise in complex anatomies, but practical implementation is limited by training and equipment availability.

## Limitations:

While in vivo studies (*e.g.*, PIPS, EndoVac) show antimicrobial efficacy, comprehensive research on long-term effects, material precipitates, or instrument interactions is lacking [25]. Many technologies remain theoretical, with insufficient evidence of clinical success across all aspects, necessitating cautious endorsement until further validated.

## Obturation strategies for conservatively prepared canals:

Minimally invasive endodontics (MIE) focuses on preserving critical tooth structures, particularly in the pericervical region, to enhance long-term tooth survival. While canal shaping is intentionally limited in MIE, effective obturation remains essential. This paper outlines evidence-based strategies adapted for conservatively prepared canals.

[1] Single-cone technique with bio-ceramic sealers: This technique aligns well with MIE due to its minimal mechanical pressure and compatibility with narrow canal preparations. Bio-ceramic sealers are bioactive, dimensionally stable and penetrate dentinal tubules effectively, forming a hydraulic seal with the canal walls [26].

[2] Ultrasonic activation of sealers: Ultrasonics enhances sealer distribution and penetration, reducing voids and improving adaptation, especially in canals with reduced mechanical shaping [26, 27].

[3] Hydraulic condensation: Cold hydraulic condensation using bio-ceramic sealers and minimal vertical force supports MIE goals by decreasing fracture risk while ensuring a dense Obturation.

[4] Advanced irrigation techniques: Since mechanical debridement is reduced, adequate irrigation becomes vital. Techniques like ultrasonic or laser activation improve smear layer removal and sealer adhesion [28].

[5] Bio-ceramic materials and MTA: MTA and other bio-ceramics are ideal in cases where apical plugs or perforation repairs are needed. Their sealing ability and regenerative properties make them suitable for MIE cases [29].

[6] Micro-CT evaluation: Micro-CT imaging has validated the effectiveness of conservative obturation strategies, showing that techniques like the single-cone method can achieve fills comparable to traditional approaches [28].

[7] Thermoplasticized gutta-percha (with caution): Using controlled heat, modified thermoplastic techniques can be adapted to MIE to enhance apical sealing without excessive pressure. However, caution is advised due to thinner canal walls.

[8] 3D-printed guides for precise delivery: Endodontic guides derived from CBCT and intraoral scans facilitate accurate access and obturation, especially in calcified or curved canals, while preserving surrounding dentin [30].

[9] Sealer-only monoblock techniques: Bioceramic sealers applied via a single-cone (sealer-only) technique can serve as the primary obturating material in narrow or fragile canals. Such approaches enhance root reinforcement through dentin bonding and biomineralization, achieving effective seal integrity [31].

[10] CBCT in obturation planning: CBCT enables detailed evaluation of canal anatomy, apical limits and potential complications. It aids in both the planning and execution of obturation in complex anatomies [32].

## Restorative considerations and tooth preservation:

Minimally invasive (MI) endodontics represents a transformative approach to root canal therapy that prioritizes the preservation of natural tooth structure while ensuring durable restorative outcomes. Combining precise endodontic techniques and biologically driven restorative strategies are essential for long-term clinical success. This approach minimizes biomechanical risks and Enhances tooth longevity [33]. Recent advancements in adhesive dentistry, conservative access methods, restorative materials and bioactive technologies have redefined restorative considerations, establishing MI endodontics as a cornerstone of modern dental practice.

## The paradigm shift in restorative dentistry:

Adhesive dentistry has revolutionized tooth preservation by allowing ultraconservative preparations that eliminate the need for aggressive mechanical retention [34]. Traditional protocols often sacrifice 2-3 mm of sound dentin to achieve a ferrule effect, which weakens endodontically treated teeth. Such treatments can lose 5-10% of fracture resistance due to access cavity preparation [35]. Modern adhesive bonding techniques, which utilize acid-etch methods; achieve comparable retention while preserving vital dentin, thereby reducing fracture risk by up to 30% in posterior teeth. Recent advancements, like self-adhering restoratives and nanohybrid composites, further enhance bond strength, with shear bond values exceeding 25 MPa, supporting MI principles [34].

## Conservative access and its impact:

Conservative access designs, such as ninja access cavities and truss designs reduce dentin removal by 40-60% compared to traditional methods. Finite element analysis shows that these designs decrease cuspal flexure by 25-30% and maintain 90% of the original tooth stiffness, lowering the risk of catastrophic fractures by 35% in premolars [35]. Integrating 3D-guided endodontics, using cone-beam computed tomography (CBCT) and dynamic navigation, reduces iatrogenic errors by 50% in calcified canals, enhancing precision [36]. Recent studies indicate that truss access cavities, combined with digital workflows, improve procedural accuracy by 20%, further preserving structural integrity [33].

## Post-Endodontic restoration strategies:

## Direct restorations:

Direct restorations focus on conservation and biomechanical compatibility. Fiber-reinforced composite (FRC) posts, with an elastic modulus of 4.5 GPa (close to dentin's 12 GPa), achieve an 83% 10-year survival rate, outperforming cast metal posts (65%). Anatomic post-space preparation using heated pluggers preserves 30% more radicular dentin than traditional drilling methods [37]. Bulk-fill flowable composites containing 78% barium glass fillers demonstrate 92% interfacial integrity, surpassing traditional composites at 74% and recent formulations with bioactive nanoparticles enhance marginal adaptation [38].

## Indirect restorations:

Endocrowns are a conservative treatment option for endodontically treated teeth, demonstrating success rates between 94% and 100%. Over a 5-year period, their estimated success rate is 77.7%, compared to 94% observed with conventional crowns [39].

## Bioactive materials and future directions:

Bioactive materials enhance MI endodontics by creating dynamic restorative interfaces. Bioceramic sealers, such as BioRoot RCS use alongside triple-action liners like TheraCal LC, release calcium and hydroxide ions for over 12 months, maintaining a pH above 10.5 to inhibit bacterial growth and achieving 300% higher sealability than traditional materials. These materials promote dentin remineralization and reduce microleakage. Emerging technologies, including 3D-printed bioceramic posts and AI-driven occlusal designs, offer unprecedented precision, with AI algorithms improving restoration fit by 15%. Future developments, such as bioactive scaffolds and regenerative endodontics, promise to enhance tooth preservation further [40].

## Conclusion:

Despite the advantages of MIE, concerns about missed canals and incomplete debridement due to ultra-conservative shaping and restricted access in complex anatomies like molars with MB2 canals persist. However, emerging regenerative therapies and the integration of AI and machine learning in preoperative planning may shift focus toward biological healing, optimize access design, reduce fracture risk and enhance endodontic success. Ultimately, MIE integrates technology, biology and biomechanics to optimize endodontic outcomes by ensuring effective disinfection and sealing while preserving healthy tooth structure.
